# Inhibition of metallo-β-lactamases in carbapenem resistant Gram negative bacilli by omeprazole and pantoprazole

**DOI:** 10.4314/ahs.v25i2.36

**Published:** 2025-06

**Authors:** Eman M Omar, Hisham A Abbas, Mona A Elsayed, Samar S Elbaramawi, Nada A Noureldin

**Affiliations:** 1 Department of Microbiology and Immunology, Faculty of Pharmacy, Zagazig University, Zagazig, Egypt; 2 Department of Medicinal Chemistry , Faculty of Pharmacy, Zagazig University, Zagazig, Egypt

**Keywords:** Meropenem, Enterobacteriaceae, *Pseudomonas aeruginosa*, Metallo-beta-lactamases, Pantoprazole, Omeprazole

## Abstract

**Background:**

Carbapenems are the most commonly used antibiotics for severe infections induced by metallo-beta-lactamases producing Enterobacteriaceae and *Pseudomonas aeruginosa*. Resistance to almost all β-lactam antibiotics, including carbapenems, is conferred by metallo-β-lactamases (MBLs).

**Objectives:**

Detection and inhibition of MBLs production as a promising approach to overcome resistance to carbapenems.

**Methods:**

160 clinical isolates of *Acinetobacter baumannii*, *Escherichia coli*, *Pseudomonas aeruginosa* and *Klebsiella pneumoniae* were tested for antimicrobial susceptibility by The disk-diffusion method. The ability of the isolates to produce MBL enzyme was detected phenotypically and genotypically by PCR. The potential ability of pantoprazole and omeprazole to inhibit metallo-β-lactamases (MBLs) was confirmed by real-time PCR.

**Results:**

Omeprazole and pantoprazole reduced the hydrolytic activities of MBLs. Both drugs had synergistic effects with meropenem. Meropenem minimum inhibitory concentration decreased in the presence of pantoprazole (2-16) folds, and omeprazole (2-32) folds. The metallo-β-lactamases genes *bla_NDM_*, *bla_IMP_* and *bla_VIM_* were downregulated by both drugs. *In silico* study showed that both drugs had reasonable binding energy and revealed that omeprazole had higher binding energy and chelating activity of zinc ions of the enzymes.

**Conclusion:**

The combination between meropenem and omeprazole or pantoprazole could be useful for treating infections caused by MBLs producing bacteria.

## Introduction

It is believed that the multidrug-resistant isolates of the ESKAPE pathogens (*Klebsiella pneumoniae*, *Enterococcus faecium*, *Pseudomonas aeruginosa*, *Staphylococcus aureus*, *Acinetobacter baumannii* and *Enterobacter spp*) cause serious nosocomial and community infections all over the world^1^. *Acinetobacter baumannii* is regarded as a common opportunistic pathogen, especially in cases of burns. Infections after burn injuries are a major cause of death. It is a significant cause of healthcare-associated infections (HCAIs), which can be fatal. These infections include wounds, urinary tract infections, meningitis, pneumonia and bacteremia^2^.

In healthy individuals, *Klebsiella pneumoniae* can live “in the nose, throat, intestines and skin”. It can, however, result in a lot of infections in patients, most commonly urinary tract infections, wound infections, pneumonia and soft tissue illnesses. Newborns, the elderly and people with compromised immune systems are among the most susceptible individuals. Furthermore, a significant percentage of serious infections that occur outside of medical facilities, including meningitis, pneumonia, and liver abscesses are caused by it^3^.

*Pseudomonas aeruginosa* is frequently found in healthy people's normal flora. However, it can also act as an opportunistic pathogen, leading to various illnesses in humans, including burns, urinary tract infections, septicemia and respiratory tract infections^4^. It is frequently obtained in medical settings, where it can lead to serious illnesses, such as urinary tract infections (UTIs), in immunocompromised people^5^.

One of the most frequent bacteria linked to urinary tract infections is *Escherichia coli*, which also plays a significant role in community-acquired illnesses including gastroenteritis, meningitis, cholecystitis, cholangitis, pneumonia, wound infections, and septicemia can result from it^6^. Because of the danger of resistant types of microorganisms, antibiotic resistance in bacteria has become a significant public health concern in recent years. The production of new antimicrobial agents is very slow^7^. When treating potentially fatal bacterial infections, β-lactam antibiotics continue to be the most important kind of medication. However, there is an increasing risk to their efficacy due to the growth of microorganisms that are resistant to drugs. The generation of β-lactamases, which catalyze the hydrolysis of the vital β-lactam ring, is the most frequent mechanism for β-lactam resistance^8^.

The primary cause of carbapenem-resistance is the synthesis of different kinds of carbapenemases. Based on amino acid sequences and molecular structure (ambler classes) there are four types of β-lactamases, which are identified as A, B, C, and D. Certain members of the A, C, and D classes of enzymes are inhibited by substances including avibactam, tazobactam, sulbactam, and clavulanic acid. These enzymes rely on an active site serine for catalysis. The Class B metallo-β-lactamases, differ structurally from Class A, C, and D enzymes. They need one or two zinc ions to induce an enzyme's reaction, and they are not inhibited by approved therapeutics^7^,^8^. With the exception of monobactams, all known β-lactam antibiotics are cleaved by MBLs, which are also resistant to beta-lactamase inhibitors and are not responsive to any serine-β-lactamase inhibitors. However, they can be inhibited by metal ion chelators, including most common MBLs families, which include active on imipenem (IMP), Verona integron-encoded MBLs (VIM) and New Delhi metallo-β-lactamase (NDM)^7^.

The use of MBL inhibitors is one of the basic mechanisms of the current antibiotic resistance treatment approaches. The two main sources of acquired MBLs are *Pseudomonas aeruginosa* and Enterobacteriaceae resistant to carbapenem. A common strategy to enhance the efficacy of β-lactam antibiotics is using inhibitors of metallo β-lactamases, which are compounds that can attach to the enzyme's active site. The purpose of this binding activity is to prevent the antibiotic hydrolysis by the enzyme^9^. Proton pump inhibitors (PPI), such as omeprazole and pantoprazole, work by permanently attaching to the hydrogen pumps in the stomach, reducing the amount of gastric acid produced^13^. They have a wide range of biological and antimicrobial properties. In addition to their ability to suppress acid release, they have demonstrated to combat against *Helicobacter pylori* due to its antibacterial activity *in vitro*^10^. The principle behind these inhibitors omeprazole and pantoprazole is the effective chelation of zinc cofactor of MBLs because of the previously reported metal chelating activity for them^11^-^13^. The aim of this work is the Investigation of new agents that can block MBLs in tested bacteria by screening the synergism between metallo-β-lactamase inhibitors and meropenems.

## Materials and methods

### Bacterial isolates

A total of 160 clinical isolates were refreshed from the Microbiology and Immunology Department's stock culture collection at Zagazig University's Faculty of Pharmacy. The clinical samples consisted of pus, urine, sputum, blood and urinary catheters. Bacterial isolates sources were identified in table 1.

**Table 1:** Distribution of bacterial isolates

Name of bacteria	Number of isolates recovered from				
	Blood	urine	pus	wound	sputum
***K. pneumoniae***	5	12	8	-	25
***E. coli***	-	50	-	-	-
***P. aeruginosa***	12	8	10	15	5
***A. baumannii***	5	2	-	3	-

### Media and Chemicals

Muller Hinton broth, tryptone agar media, MacConkey agar media, agar -agar media and nutrient broth media were purchased from Oxoid (UK). Antibiotics disks were ceftriaxone (CRO, 30 µg), ampicillin (AMP, 10 µg) , ampicillin-sulbactam (SAM, 10/10 µg) , cefepime (FEP, 30 µg), piperacillin (PIP, 100 µg) , piperacillin-tazobactam (TZP,100/10 µg), gentamicin (GN, 10 µg), Imipenem (iPM, 10 µg), colistin (CT, 10 µg), ceftazidime (CAZ, 30 µg), aztreonam (ATM, 30 µg) and azithromycin (AZM, 15 µg) were purchased from Oxoid (UK). Ethylene-diamine tetra-acetic acid (EDTA) at PH 8 and di methyl formamide were purchased from Sigma Chemical Co. (St. Louis, MO, USA). Omeprazole and pantoprazole were a gift from Delta pharma company, Tenth of Ramadan city, Egypt.

### Antimicrobial susceptibility testing

Bacterial isolates of *Acinetobacter baumannii*, *Klebsiella pneumoniae*, *Pseudomonas aeruginosa* and *E. coli* were exposed to susceptibility testing against various β-lactams using the disk diffusion method according to CLSI (Clinical and Laboratory Standards Institute) guidelines^14^. Five millilites of sterile Mueller Hinton broth (MHB) were inoculated with four colonies from each isolate, and the mixture was then incubated until the turbidity level was equal to a 0.5 McFarland standard. After that, the broth cultures were diluted in broth 1:200 until reaching to a density of 105–106 cells/ml. After modifying the inoculums' density for fifteen minutes, a sterile cotton swab was immersed in the bacterial suspension. After that, the extra suspension was removed by repeatedly rotating the cotton swab in the tube's inner wall above the fluid's level. Using forceps, the inoculation from the inoculated plates was gently pressed into the outside of a desiccated Mueller Hinton agar plate. After incubation period in the incubator at 37 °C, the plates were turned over and kept there. After examining the plates closely, the total inhibition zones were measured in millimeters. According to CLSI criteria, the results were interpreted^15^.

### Combined EDTA Disk Synergy test for detection of MBL producers

Metallo-β-Lactamases were identified by use of the Combined EDTA Disk Synergy test. The increase of imipenem inhibition zones in the presence of EDTA (pH 8) served as the basis for the detection of MBLs in this method. A Mueller-Hinton agar plate was coated with a liquid culture of carbapenem-resistant isolates that had been standardized to a turbidity equal to a 0.5 McFarland standard over the course of an overnight. The plate was coated with two imipenem (10 µg) discs, and one of them received 10 µl of EDTA solution, resulting in a target concentration of 750 After that, the plates were put in an incubator and allowed to incubate for 18 hours. An imipenem-EDTA disc was considered to be an MBL producer if its inhibition zone was at least 7 mm bigger than that of an imipenem disc alone^16^.

### Genotypic detection of metallo-Beta Lactamase genes by Polymerase Chain Reaction (PCR)

In order to confirm the Prescence of genes encoding MBL in tested strains, PCR was employed. PCR aimed to determine the presence of *bla_NDM_*, *bla_IMP_*, and *bla_VIM_* genes using the primers in table (2). Firstly we extract gDNA via taking a pure colony using a sterile pipette tip from night culture in Mueller-Hinton agar plates and placed in 20 µL distilled water for all the tested strains, After 10 seconds of vortexing, the mixture was incubated for five minutes at 98°C, followed by centrifugation to the lysate then the supernatant was separated, diluted (1:3 dilution ratio) with distilled water, and then PCR analysis was carried out. A total volume of 50µL was obtained by mixing 10.0µL of DNA template, 2.0µL of each of the forward and reverse primers, nuclease-free water, and 25µL of 2x PCR master mix from AB gene UK for each PCR mixture. Germany's Operon Biotechnologies GmbH Biocompass in Cologne designed the primers. Initial denaturation at 95 °C for 5 minutes was part of the cycling parameters. Following that, there were thirty cycles of denaturation for one minute at 95 °C, annealing for one minute at 52 °C, and extension for one minute at 68 °C. The final extension was run at 68 °C for five minutes. After electrophoresis , the PCR results on 2.0% agarose gels stained with 1% ethidium bromide, photographs were taken^5^.

**Table 2:** Primers used in PCR amplifications

Name of gene bacteria		Primer Sequence	size	Reference
***E. coli, and K. pneumoniae***	*bla_NDM_*	F = 5′-GCATAAGTCGCAATCCCCG -3′ R =5′-CTTCCTATCTCGACATGCCC-3′	237 bp	(7)
	*bla_IMP_*	F = 5′-GGAATAGAGTGGCTTAAYTC-3′ R=5TCGGTTTAAYAAAACAACCACC-3′	232 bp	
	*bla_VIM_*	F = 5′-GATGGTGTTTGGTCGCATA-3′ R =5′-CGAATGCGCAGCACCAG-3′	390 bp	
***A. baumannii***	*bla_NDM_*	F=5′-GGTTTGGCGATCTGGTTTTC-3′ R =5′-CGGAATGGCTCATCACGATC-3′	627 bp	(2)
	*bla_IMP_*	F=5′-TGAGCAAGTTATCTGTATTC-3′ R = 5′-TTAGTTGCTTGGTTTTGATG3′	740 bp	
	*bla_VIM_*	F=5′-AAAGTTATGCCGCACTCACC-3′ R = 5′-TGCAACTTCATGTTATGCCG-3	815 bp	
***P. aeruginosa***	*bla_IMPA_*	F: 5′-AAAGACGGTAAGGTTCAA-3′ R: 5′-CGCCTGCTCTAATGTAAG-3′	318 bp	(17)
	*bla_IMPB_*	F: 5′-ACATTTCCATAGCGACAG-3′ R: 5′-TGTTCCCATGTACGTTTC-3′	408 bp	
	*bla_VIMA_*	F: 5′-AGTGGTGAGTATCCGACAG-3′ R: 5′-ATGAAAGTGCGTGGAGAC-3′	261 bp	

### Determination of MIC of meropenem and tested drugs against MBL isolates

Following CLSI guidelines, the agar dilution method was used to determine the minimum inhibitory concentrations (MIC) of meropenem and the tested drugs (7). Various concentrations of meropenem and tested drugs were formulated on nutrient agar plate. The following concentrations were prepared: (32-2048) µg/ml for meropenem and (250-8000) µg/ml for pantoprazole and omeprazole. Five milliliters of nutrient broth were added to an agar plate containing a colony from each sample. Following the incubation period at 37°C, it was necessary to dilute the cultures in sterile saline and control their turbidity to be equal to 0.5 McFarland standard. In sterile saline, this solution was further diluted 1:10. The final inoculum, approximately 104 colony-forming units (cfu) per spot, was obtained by applying two µl of the bacterial suspension over the surface of Muller agar plates using a calibrated micropipette. The plates were placed in the incubator at 37 °C for 18 hours, during which time growth was detected. The lowest concentration at which observable growth was inhibited was identified as the minimum inhibitory concentration) MIC.

### Effect of pantoprazole and omeprazole on bacterial cell viability

The effect of the drug on bacterial growth was performed in prescence and absence of sub-MIC (¼ MIC) (2000 ug/ml) of pantoprazole and omeprazole. Overnight culture in Mueller-Hinton broth was prepared and the culture was diluted to have a turbidity equal to 0.5 McFarland standard of bacterial isolates followed by dilution to 1/100 concentration in muller broth with sub-MIC of pantoprazole divide Mueller-Hinton tubes into control and treated by drugs sub-MIC then put it in incubator after 24 hrs we measure optical density at 600nm using spectrofluorometer (Biotek, USA)^18^.

### Carbapenemase inhibition assay

A loopful of bacteria was added to ten milliliters of Mueller-Hinton broth for each bacterial isolate, and the mixture was then cultured in an orbital shaking incubator for eighteen hours. Followed by centrifugation to the suspension at 15,000 g for ten minutes in order to extract the pellets. Next, 50 µM ZnSO4 was added to 500 µl of phosphate buffer (100 mM, pH 7.0) in a microcentrifuge tube, and the pellets were mixed again. The tube was then sonicated for 1.5 minutes at 40 W using a 0.5 s pulse using an Ultrasonic System UP100H from Hielscher–Ultrasound Technology, Teltow, Germany. Following a 10-minute centrifugation at 15,000 g at 4 °C for 10 minutes, the bacterial suspensions were used to measure the amount of meropenem hydrolysis activity with and without 2 mg/ml of pantoprazole and omeprazole at 297 nm using UV-Vis spectrophotometer (synergy HT BioTek). A 96-well microtitre plate was filled with hundred µl aliquots, and the plate was incubated for 30 minutes with (2000-1000-500) µg/ml pantoprazole and omeprazole. After adding 500 µg/ml of meropenem, the solutions were incubated at 37 °C for one hour. At 297 nm, the drug-containing solutions' absorbances were measured. Using the following formula, the percentage of inhibition of meropenem hydrolysis was determined: % of inhibition = [(OD of treated - OD control)/OD of treated] x 100^19^.

### Investigation of the synergy between meropenem and tested drugs

In order to investigate the synergy between meropenem and tested drugs, MIC of meropenem was determined in the presence of sub MICs (1/4 , 1/8 and 1/16 MIC) of pantoprazole and omeprazole by the agar dilution method^7^.

### RNA isolation and quantitative real time PCR (qRT-PCR)

The qRT-PCR was employed to evaluate the effect of omeprazole and pantoprazole on the MBL genes expression (*bla_NDM_*, *bla_VIM_* and *bla_IMP_*). In summary, 0.5 milliliters of the bacterial suspension that is 0.5 McFarland concentration was mixed with 5 milliliters of Luria-Betani (LB) broth. The cells were harvested after 18 hours of incubation at 37 °C. Thermo Fisher Scientific Inc, Germany's GeneJET RNA purification kit was used for extraction the total RNA in accordance with the manufacturer's recommendations . To generate complementary DNA from total RNA (2µg) using random hexamer primers (ThermoFisher Scientific, USA) in a final reaction volume of 20µL, the manufacturer's instructions for SuperScriptTM II RT (Invitrogen™, California, USA) were adhered to. A 96-well Agilent Stratagene Mx3005P qPCR Cycler System 5 Color was used to quantify gene transcripts. The PowerUpTM SYBRTM Green Master Mix was provided by Applied Biosystems and Thermo Fisher Scientific. The primers wrRNA as a housekeeping gene, fold changes in the expression of the examined genes were measured^20^.

### Molecular docking study

Structures of omeprazole and pantoprazole were retrieved from the PubChem database (https://pubchem.ncbi.nlm.nih.gov/ accessed on 4 February 2024) as canonical SMILES. Crystal structures of *K. pneumoniae* NDM-1 metallo-beta-lactamase (PDB: 5NBK), *P. aeruginosa* IMP-13 metallo-beta-lactamase (PDB: 6S0H) and E. coli NDM-5 metallo-beta-lactamase (PDB: 4TZE)^21^,^22^. Which were obtained from the RCSB Protein Data Bank (https://www.rcsb.org/ accessed on 4 February 2024)^23^. *A. baumannii* NDM-1 metallo-beta-lactamase (Uniprot Entry: F8UNN7_ACIBA, www.uniprot.org accessed on 4 February 2024) has no resolved crystal structure, so SWISS-MODEL prediction was utilized to build a homology model for docking studies^24^. The OpenEye software was used to perform docking studies^25^. A 3D structures for both omeperazole and pantoprazole were obtained using Omega classic^26^,^27^, 100 conformation for each compound were created. The protein structures were prepared using make_receptor 4.3.0.3 module^28^. While, the FRED module was used to perform docking using Chemguess4 scoring function^28^,^29^. Both Vida 5.0.5.3^30^ and PyMOL softwares were used to visualize the 10 generated docking poses^31^.

## Results

### Susceptibility of the tested bacterial isolates to different classes of antimicrobial agents

**Figure 1:**
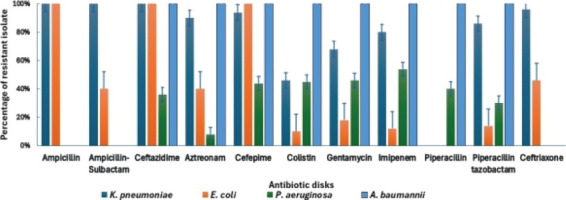
Percentage of resistant bacterial isolates to antibiotics

The tested 160 bacterial isolates showed high rate of Multidrug resistance and imipenem resistance in Table 3. The high multidrug resistance and Imipenem resistance were found in *Acinetobacter baumannii* isolates followed by *Klebsiella pneumoniae*, *Pseudomonas aeruginosa* then Escherichia coli isolates in figure 1.

**Table 3:** Percentage of prevalence of MBL genes in bacterial isolates

Name of gene bacteria		Percentage of prevalence of MBL genes	
***E. coli* and *K. pneumoniae***	*bla^NDM^*	41%	
	*bla^IMP^*		0%
	*bla^VIM^*		12%
***A. baumannii***	*bla^NDM^*	*71%*	
	*bla^IMP^*	0%	
	*bla^VIM^*	14%	
***P. aeruginosa***	*bla^IMPA^*		0%
	*bla^IMPB^*	0%	
	*bla^VIMA^*	50%	

### Production of Metallo-beta-lactamase (MBL) by the tested isolates

Detection of MβL enzyme was done for the 83 imipenem resistant isolates . Forty one percent of isolates produced MβL enzyme according to (CDDT) and (59%) isolates were negative for this test. The highest rate of MBL production in Acinetobacter baumannii but the lowest rate in *Escherichia coli*. Thirty three percent of *Escherichia coli*, (37%) of *Klebsiella pneumoniae* and *Pseudomonas aeruginosa* and (70%) of Acinetobacter baumannii isolates.

### Detection of MBL genes among MBL-producers

PCR was performed on 34 imipenem resistant isolates for detection the genes encoding for Metallo-beta-lactamase (MBL). *Bla_NDM_* gene in PCR products of was the most prevalent gene while *bla_IMP_* genes in PCR products was the least prevalent gene.

### Antibacterial activity of tested drugs on cell viability for pantoprazole and omeprazole

**Figure 3:**
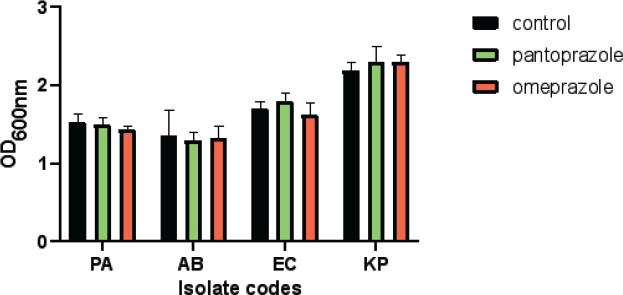
Antibacterial activity of tested drugs on cell viability for different bacterial isolates

To ensure that sub-MIC of tested drugs had no effect of bacterial growth the cell viability shown in Figure 3 was assessed in Prescence of this concentration (1/4 MIC). There is no significant difference in absorbance between control and treated isolates.

### Determination of meropenem MIC in presence of sub-MIC of pantoprazole and omeprazole

In order to investigate synergy between meropenem and tested drugs MIC of meropenem was redetermined in presence sub-MIC shown in table 4. The combination of sub-MIC of pantoprazole and omeprazole exerted a significant reduction in meropenem MIC (2 to 32 folds) for omeprazole and (2-16) fold for pantoprazole . Omeprazole is more active than pantoprazole.

**Table 4:** The effect of sub-MIC of pantoprazole and omeprazole on susceptibility against meropenem

Isolate's No	MEM MC(ug/ml)	MEM + omeprazole			MEM + pantoprazole		
		1/4 MIC	1/8 MIC	1/16 MIC	1/4 MIC	1/8 MIC	1/16 MIC
**KP1**	256	32	64	128	32	64	64
**KP2**	256	32	128	128	64	128	128
**KP3**	1024	128	256	256	256	256	512
**KP4**	64	16	16	32	16	32	32
**KP5**	128	32	64	64	32	64	64
**EC1**	32	1	16	16	4	8	16
**EC2**	32	1	8	16	4	8	8
**AB1**	128	16	64	64	32	32	64
**AB2**	64	16	32	32	16	16	32
**AB3**	256	16	32	64	64	128	128
**AB4**	256	64	64	128	64	64	128
**AB5**	32	1	4	8	4	8	16
**PA1**	1024	256	512	512	512	512	512
**PA2**	2048	256	512	1024	512	1024	1024
**PA3**	2048	512	512	1024	512	1024	1024
**PA4**	1024	64	256	512	128	256	256
**PA5**	1024	128	256	256	256	256	512

MEM : meropenem

In *Klebsiella pneumoniae* and in *Pseudomonas aeruginosa* meropenem MIC was reduced (2-16) fold for omeprazole and (2-8) fold for pantoprazole. In *Escherichia coli* meropenem MIC was reduced (32) fold for omeprazole and only (8) fold for pantoprazole. In *Acinetobacter baumannii* meropenem MIC was reduced (2-32) fold for omeprazole and (2-8) fold for pantoprazole.

### The tested drugs inhibited MBL production

**Figure 4:**
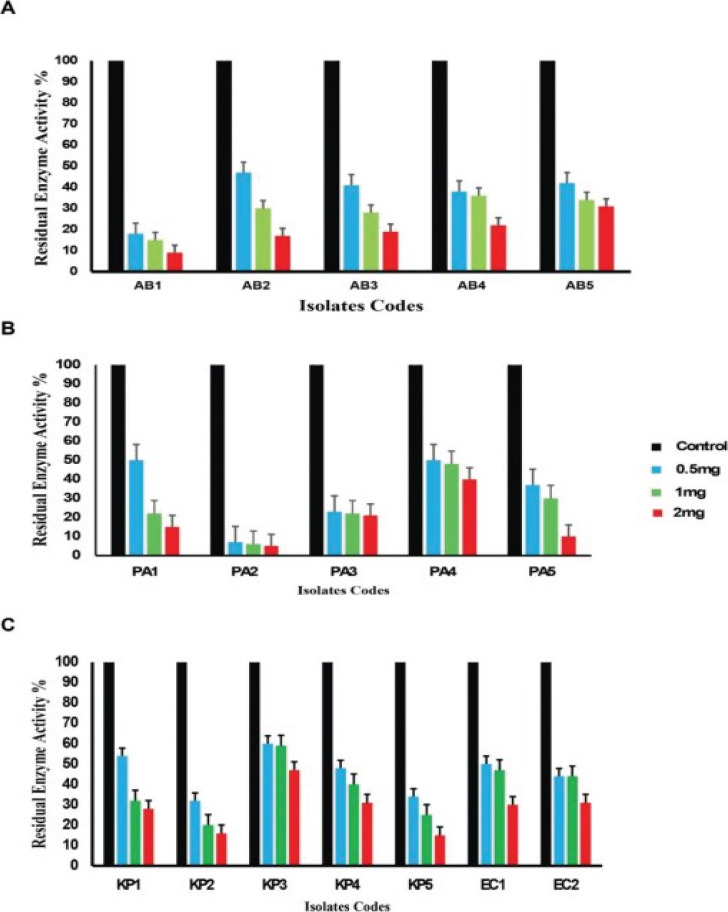
Carbapenemase inhibition assay with pantoprazole for bacterial isolates

**Figure 5:**
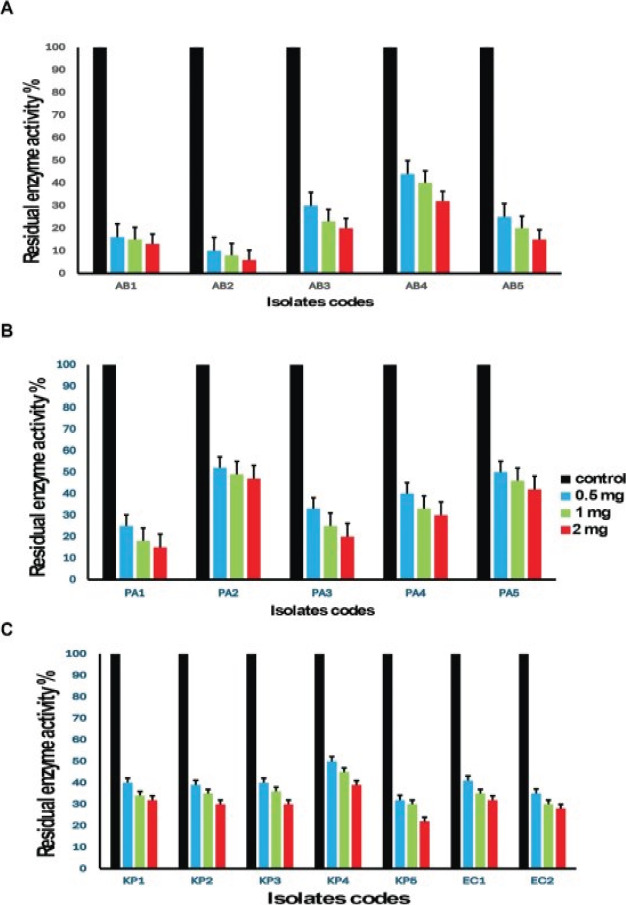
Carbapenemase inhibition assay with omeprazole for bacterial isolates

Carbapenemase inhibition assay with pantoprazole and omeprazole demonstrated significant inhibition of meropenem hydrolysis as shown in the figures 4,5. Omeprazole was more active than pantoprazole. In *Acinetobacter baumannii* isolates pantoprazole inhibition of meropenem hydrolysis (53%-91%) while in omeprazole the inhibition from (56%-94%). In *Pseudomonas aeruginosa* isolates omeprazole (34%-96%) while in pantoprazole the inhibition from (56%-94%). In *K. pneumoniae* isolates omeprazole (40%-84%) while in pantoprazole the inhibition from (47%-78%. In *Escherichia coli* isolates omeprazole (50%-70%) while in pantoprazole the inhibition (50%-63%)

### Omeprazole and pantoprazole downregulated MBL genes

**Figure 6:**
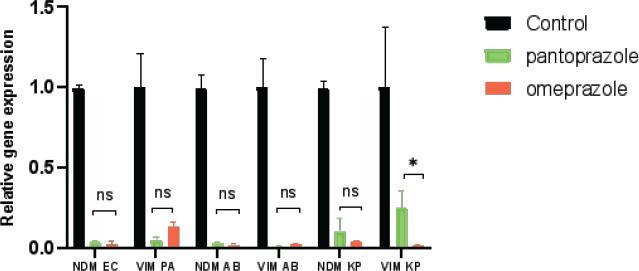
pantoprazole and omeprazole decreased the expression of MBL genes as revealed by qRT-PCR

The qRT-PCR was performed to evaluate the effect of pantoprazole and omeprazole on the genes encoding carbapenemase expression level in bacterial isolates. Results showed that the tested genes' expression was much lower in the treated bacteria than in the untreated cells. (2000 µg/mL) of the two drugs decreased the expression of **bla_NDM_**, *bla_IMP_* and *bla_VIM_* (Fig 6). Omeprazole and pantoprazole significantly decreased the expression of MBL genes. Omeprazole decreased the expression of both *bla_NDM_, bla_VIM_* of K. pneumoniae more than pantoprazole, while pantoprazole decreased the expression of *bla*_*VIM*_ of *P. aeruginosa* more than omeprazole. Both decreased the expression of both *bla_NDM_*, *bla_VIM_ of A. baumannii and bla_NDM_* of *E. coli*.

### Molecular docking

The molecular docking study was performed to gain insights into the interactions of omeprazole and pantoprazole on *K. pneumoniae*, *P. aeruginosa*, *E. coli* and *A. baumannii metallo-beta* lactamase enzymes. Molecular docking of both omeprazole and pantoprazole showed reasonable binding energy (S score = -8.71 Kcal/mol, -8.08 Kcal/mol, respectively) within K. pneumoniae NDM-1 metallo-beta-lactamase enzyme and showed numerous interactions with the amino acid residues (Table 5). Pyridinyl ring and the sulfinyl moiety of omeprazole formed ionic interactions with one of the two zinc metal ions in the active site, while the pyridinyl ring formed a hydrogen bond interaction with Asp124. Whereas the benzimidazole ring formed hydrogen bond with Lys211. The same interactions were observed with pantoprazole.

**Table 5:** Docking results for omeprazole and pantoprazole with *K. pneumoniae*, *P. aeruginosa*, *E. coli* and *A. baumannii metallo-beta* lactamases

Bacteria	Drug	S Score (Kcal/mol)	Ionic Interactions	Hydrogen Bonds
*K. pneumoniae*	Omeprazole	-8.71	Zn^+2^	Asp124, Lys211
	Pantoprazole	-8.08	Zn^+2^	Asp124, Lys211
*P. aeruginosa*	Omeprazole	-11.76	Zn^+2^	Lys161, Asp167
	Pantoprazole	-10.71	Zn^+2^	Lys161, Asp167
*E. coli*	Omeprazole	-9.7	Zn^+2^	Asp124, Lys211
	Pantoprazole	-8.71	Zn^+2^	Asp124, Lys211
*A. baumannii*	Omeprazole	-7.2	Zn^+2^	Gln123, Asn220
	Pantoprazole	-7.57	Zn^+2^	Asn220

Within the active site of *P. aeruginosa* IMP-13 metallo-beta-lactamase, omeprazole and pantoprazole exhibited numerous interactions with energy scores of -11.71 and -10.76 Kcal/mol, respectively (Table 5). Pyridinyl ring of omeprazole showed ionic interaction with one of the two zinc metal ions, whereas sulfinyl moiety formed two hydrogen bond interactions with both Lys161 and Asn167. The same interactions were observed with pantoprazole Within the active site of *E. coli* NDM-5 metallo-beta-lactamase, omeprazole showed energy score -9.70 Kcal/mol. Benzimidazole ring and sulfinyl moiety exhibited hydrogen bonds with Lys211. Additionally, pyridinyl rig of omeprazole showed an ionic interaction of zinc metal and a hydrogen bond with Asp124. For pantoprazole the energy score was -8.71 Kcal/mol, the drug showed the same binding interaction as omeprazole, moreover there was an extra ionic interaction between sulfinyl moiety and the zinc metal ion.

Within the active site of *A. baumannii* NDM-1 metallo-beta-lactamase, methoxy substituent shows hydrogen bonding with Gln123 the same as the interaction of the pyridinyl group with Asn220. The benzimidazole ring shows an ionic interaction with zinc metal ion. For pantoprazole, sulfinyl moiety has hydrogen and ionic interactions with Lys211 and zinc metal ion respectively. The benzimidazole ring has ionic interaction with zinc metal ion.

**Figure 7:**
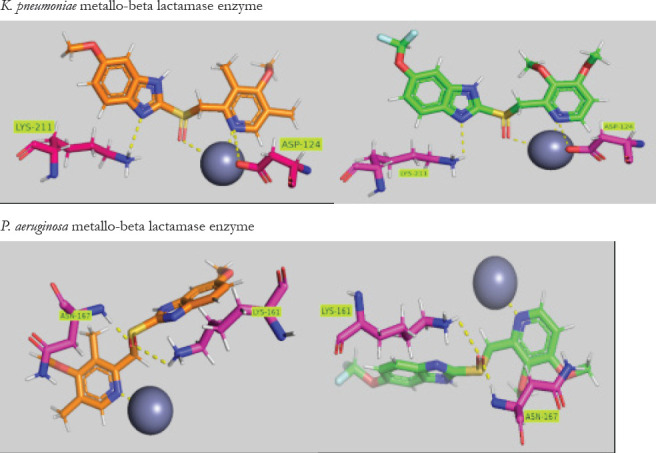

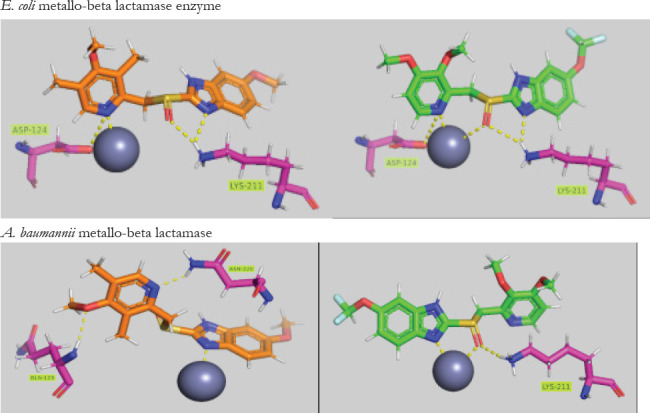
Three-dimensional drug-enzyme interactions. Omeprazole is thick orange sticks; pantoprazole is thick green sticks and amino acid residues are magneta sticks. Interactions are shown as yellow dots

Both drugs are stabilized within the active site of *K. pneumoniae*, *P. aeruginosa*, *E. coli* and *A. baumannii* metallo-beta-lactamases through numerous interactions with zinc metal and amino acid residues. These interactions are conserved to a high extent between both drugs and metallo-beta-lactamase proteins derived from different bacteria (Table 5). Figure 7 shows the interacting amino acid residues present in the active site of metallo-beta-lactamases for the targeted bacteria, proposing that omeprazole and pantoprazole would be effective inhibitors against *K. pneumoniae*, *P. aeruginosa*, *E. coli* and *A. baumannii* metallo-beta lactamases.

## Discussion

The resistance of metallo-beta-lactamases producing Enterobacteriaceae and *Pseudomonas aeruginosa* to meropenem is a significant cause for concern because they were typically resistant to most clinically available antimicrobial agents. This widespread resistance severely restricts therapeutic choices, leaving limited options for effective treatment^32^. The bacterial isolates showed high rate of MDR which ranged from 30% to 100% and high rate of imipenem resistance which ranged from 12% to 100% . A pervious study for *A. baumannii*, MDR and rate of resistance to imipenem was lower than our study^2^. Another study for *K. pneumoniae* showed the same percent of MDR (84%) and lower rate of resistance to imipenem compared to our study^33^. Compared to this study ,some studies for *E. coli* and *P. aeruginosa* isolates showed higher MDR and lower carbapenem resistance^34^,^35^.

The ability of the isolates to produce MBL enzyme was detected phenotypically by combined disk method and confirmed genotypically by PCR. In this study, PCR showed higher prevalence of *bla_VIM-1_* of *P. aeruginosa* than Tarashi et al^36^, while the other gene blaIMP was not detected in both studies. The blaNDM was detected in *A. baumannii* isolates; while in Tarashi et al none of the isolates tested positive for this gene, however, the *blaVIM-1* was found nearly within the same range^36^. Moreover, another study on *E. coli* and *K. pneumoniae* strains, showed the presence of NDM-1 as the predominant enzyme as similar to our study. However, the VIM and IMP genes were identified with higher rates^37^.

Because of the limited treatment options for MBL producing bacteria , there is a need for identification of novel MBL inhibitors. The current therapies include small number of antibiotics like tigecycline and polymyxins. However, it is difficult to treat carbapenem-resistant *K pneumoniae* with tigecycline, a broad-spectrum antibiotic of the tetracycline class, because of some limitations; the medication's low circulatory, respiratory, and urinary system concentrations. As such, it is not considered a drug of choice for the treatment of infections for urinary tract infections, pneumonia, and bacteremia^38^. The use of polymyxin B, a polypeptide antibiotic, is restricted because of its nephrotoxic adverse effects^39^. Another therapy is based on combination of β-lactam and β-lactamase inhibitors, imipenem, cilastatin and relebactam (IMI-REL), was licensed to treat complex urinary tract infections. As a β-lactamase inhibitor, relebactam has been shown to be able to efficiently inhibit a variety of β-lactamases, including class A and class C β-lactamases, such as carbapenemases. Relebactam restored imipenm's effectiveness against a range of bacteria resistant to imipenem when coupled with imipenem. To increase its overall antibacterial action, it is co-formulated with imipenem-cilastatin to create imipenem-cilastatin-relebactam^40^. The efficacy of vaborbactam, a serine β-lactamase inhibitor, when used with meropenem against Enterobacteriaceae that produce serine carbapenemase (CPE), and the meropenem activity was restored by the addition of vabrobactam, but it is inactive against class-B or D carbapenemases^41^.

Because of the limitations of antibiotic therapy, previous studies discovered some MBL inhibitors like captopril which was used to increase the susceptibility of carbapenem-resistant *K pneumoniae* (CRKP) that produce metallo-β-lactamase (MBL) to antimicrobial drugs by reversing drug resistance. In order to obtain insight into treatment approaches for drug-resistant MBL-producing bacteria, the study also aimed to enhance captopril's binding affinity for imipenemase^42^. In another study, malic acid, citric acid, ascorbic acid and ciprofloxacin in combination with imipenem or meropenem made asynergy against metallo-beta-lactamases. Their activities may be due to the metal chelation in the active site of carbapenemases^7^. Moreover, celastrol and thymol together against isolates of *K. pneumoniae* resistant to carbapenem. Thymol makes celastrol more permeable via the CRK-envelope. To restore the activity of meropenem and other β-lactams, more secure and efficient analogues could be developed using the triple combination of meropenem, celastrol, and thymol^33^.

In this study, we investigate the comparative activity of omeprazole and pantoprazole on different bacterial strains *Pseudomonas aeruginosa*, *Klebsiella pneumonia*, *Acinetobacter baumanii* and *Escherichia coli*. The two drugs were employed at sub-inhibitory concentrations, ensuring that cell growth remained unaffected, thus eliminating the possibility of any impact on metallo beta-lactamases due to interference with cell growth. Meropenem-MIC decreased by pantoprazole (2-16) folds while with omeprazole, it decreased by (2-32), while in a pervious study pantoprazole was found to have inhibitory activity against *Klebsiella pneumonia* MBL enzymes via decreasing MIC of meropenem by 4 folds^38^. Omeprazole outperformed pantoprazole in its capacity to protect meropenem from hydrolysis mediated by the carbapenemase enzyme when tested for their actions against the inhibition of carbapenem meropenem hydrolysis. Pantoprazole and omeprazole were selected due to prescence of benzimidazole nucleus that was previously reported as metal chelating activity (11-13). Zinc ions are necessary for metallo beta-lactamases to function and are found in their active sites. The action of metallo beta-lactamases can therefore be inhibited by zinc chelation. Omeprazole showed higher activity than pantoprazole . The potential strong interaction between pantoprazole and omeprazole with zinc ions within the active site of the metallo-beta-lactamase enzyme was demonstrated by an *in-silico* investigation.

The binding energy scores for omeprazole are lower than that of pantoprazole, indicating that the binding affinity of omeprazole is higher than that of pantoprazole. These results match the *in vitro* results^22^.

To further confirm the inhibitory activities of the two drugs against MBL enzymes, real time PCR was employed and showed the downregulation of the examined genes in treated bacteria relative to untreated cells. omeprazole decreased the expression of both *bla_NDM_*, *bla_VIM_* of *K. pneumoniae* more than pantoprazole while pantoprazole decreased the expression of *bla_VIM_* of *P. aeruginosa* more than omeprazole. Both decreased the expression of both *bla_NDM_*, *bla_VIM_* of A. baumannii and bla_NDM_ of *E. coli*. In a previous study pantoprazole downregulated the expression of both *bla_VIM_* and *bla_NDM_* for *K pneumonia* less than our study^38^.

## Conclusion

Pantoprazole and omeprazole can be used when combined with meropenem to treat server resistant infections because they reversed drug resistance in MBL-producing bacterial isolates to restore the sensitivity of the bacterial isolates to antimicrobial drugs via the potential chelation of zinc cofactor of MBLs.

## Figures and Tables

**Figure 2:**
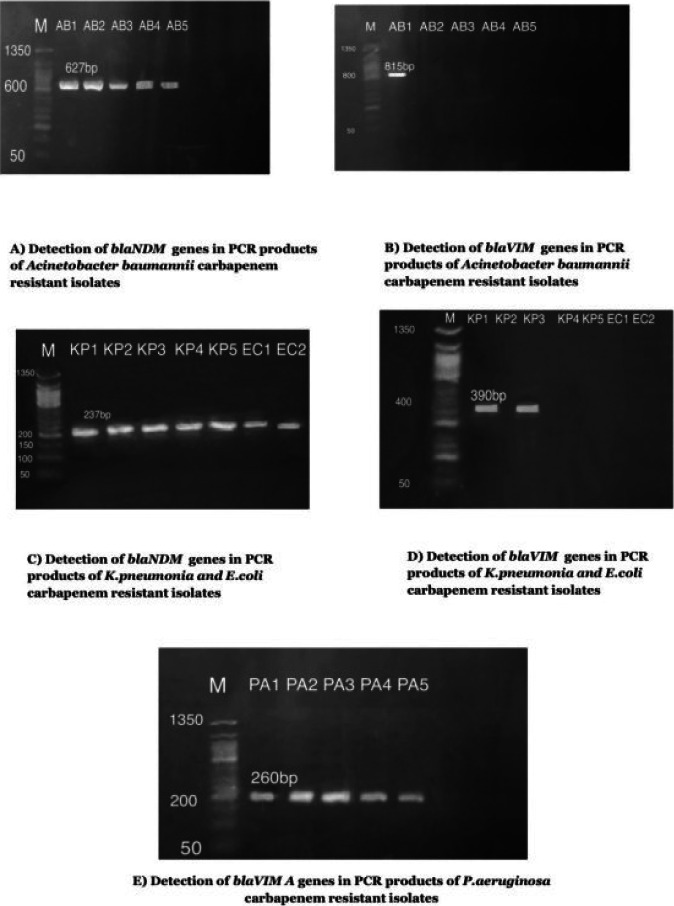
Detection of MBL genes among MBL-producers bacterial isolates, *KP: K. pneumoniae; EC: E. coli; PA:* P. *aeruginosa; AB: A. baumannii*

## References

[1] Christiana AE, Theolyn C, S'thebe WN, Yamkela D, Saheed S (2024). ESKAPE pathogens and associated quorum sensing systems–New targets for novel antimicrobials development. Health Sciences Review.

[2] Golafshani FB, Kaboosi H, Armaki MT, Ghadikolaii FP, Fattahi E (2019). Molecular investigation of integron types and imipenem-resistance encoded genes in *Acinetobacter baumannii* strains isolated from burns patients in Iran. Gene Reports.

[3] Moya C, Maicas S (2020). Antimicrobial resistance in *Klebsiella pneumoniae* strains: mechanisms and outbreaks. Proceedings.

[4] Kadry A, Ezz M, A Abbas H (2018). Phenotypic and genotypic characterization of *Pseudomonas aeruginosa* siderophores. Zagazig Journal of Pharmaceutical Sciences.

[5] Abbas HA, El-Ganiny AM, Kamel HA (2018). Phenotypic and genotypic detection of antibiotic resistance of Pseudomonas aeruginosa isolated from urinary tract infections. African Health Sciences.

[6] Collingwood JD, Wang L, Aban IB, Yarbrough AH, Boppana SB, Dangle PP (2023). Risk factors for community acquired pediatric urinary tract infection with extended-spectrum-β-lactamase *Escherichia coli*-A case-control study. Journal of Pediatric Urology.

[7] Abbas HA, Kadry AA, Shaker GH, Goda RM (2019). Impact of specific inhibitors on metallo-β-carbapenemases detected in *Escherichia coli* and *Klebsiella pneumoniae* isolates. Microbial pathogenesis.

[8] Klingler F-M, Wichelhaus TA, Frank D, Cuesta-Bernal J, El-Delik J, Müller HF (2015). Approved drugs containing thiols as inhibitors of metallo-β-lactamases: strategy to combat multidrug-resistant bacteria. Journal of Medicinal Chemistry.

[9] Vázquez-Ucha JC, Arca-Suárez J, Bou G, Beceiro A (2020). New carbapenemase inhibitors: clearing the way for the β-lactams. International Journal of Molecular Sciences.

[10] Sjöström J, Fryklund J, Kühler T, Larsson H (1996). In vitro antibacterial activity of omeprazole and its selectivity for *Helicobacter* spp. are dependent on incubation conditions. Antimicrobial Agents and Chemotherapy.

[11] Hegazy W, Issa Y, El-Sayed W, Ahmed H (2016). Panantoprazole sodium sesquihydrate complexes: Synthesis, characterization, potentiometric determination and DNA interaction. Oriental Journal of Chemistry.

[12] Chkirate K, Essassi EM (2022). Pyrazole and Benzimidazole Derivatives: Chelating Properties Towards Metals Ions and their Applications. Current Organic Chemistry.

[13] Salama F, El-Abasawy N, Razeq SA, Ismail M, Fouad M (2003). Validation of the spectrophotometric determination of omeprazole and pantoprazole sodium via their metal chelates. Journal of Pharmaceutical and Biomedical Analysis.

[14] Wayne P (2018). CLSI Performance Standards for Antimicrobial Susceptibility Testing. CLSI supplement M100. Vol. 28^th^ ed.

[15] Moulana Z, Babazadeh A, Eslamdost Z, Shokri M, Ebrahimpour S (2020). Phenotypic and genotypic detection of metallo-beta-lactamases in Carbapenem resistant *Acinetobacter baumannii*. Caspian Journal of Internal Medicine.

[16] Pandya NP, Prajapati SB, Mehta SJ, Kikani KM, Joshi PJ (2011). Evaluation of various methods for detection of metallo-β-lactamase (MBL) production in gram negative bacilli. Int J Biol Med Res.

[17] Yu G, Wen W, Peters BM, Liu J, Ye C, Che Y (2016). First report of novel genetic array aacA4-blaIMP-25-oxa30-catB3 and identification of novel metallo-β-lactamase gene blaIMP25: A Retrospective Study of antibiotic resistance surveillance on *Psuedomonas aeruginosa* in Guangzhou of South China, 2003–2007. Microbial Pathogenesis.

[18] Nalca Y, Jänsch L, Bredenbruch F, Geffers R, Buer J, Häussler S (2006). Quorum-sensing antagonistic activities of azithromycin in *Pseudomonas aeruginosa* PAO1: a global approach. Antimicrobial Agents and Chemotherapy.

[19] Pal A, Tripathi A (2020). Quercetin inhibits carbapenemase and efflux pump activities among carbapenem-resistant Gram-negative bacteria. Apmis.

[20] Livak KJ, Schmittgen TD (2001). Analysis of relative gene expression data using real-time quantitative PCR and the 2– ΔΔCT method. Methods.

[21] Raczynska JE, Shabalin IG, Minor W, Wlodawer A, Jaskolski M (2018). A close look onto structural models and primary ligands of metallo-β-lactamases. Drug Resistance Updates.

[22] Softley CA, Zak KM, Bostock MJ, Fino R, Zhou RX, Kolonko M (2020). Structure and molecular recognition mechanism of IMP-13 metallo-β-lactamase. Antimicrobial Agents and Chemotherapy.

[23] Dutta S, Burkhardt K, Bluhm WF, Berman HM (2005). Using the tools and resources of the RCSB protein data bank. Current Protocols in Bioinformatics.

[24] Waterhouse A, Bertoni M, Bienert S, Studer G, Tauriello G, Gumienny R (2018). SWISS-MODEL: homology modelling of protein structures and complexes. Nucleic acids research.

[25] Fred V (2006). OpenEye Scientific Software.

[26] 4. OMEGA 5.1.0.0 https://www.eyesopen.com.

[27] Hawkins PC, Skillman AG, Warren GL, Ellingson BA, Stahl MT (2010). Conformer generation with OMEGA: algorithm and validation using high quality structures from the Protein Databank and Cambridge Structural Database. Journal of Chemical Information and Modeling.

[28] OEDOCKING 4.3.1.0 https://www.eyesopen.com.

[29] McGann M (2011). FRED pose prediction and virtual screening accuracy. Journal of Chemical Information and Modelmg.

[30] Doman AJ, Perkins MV, Tommasi S, Mangoni AA, Nair PC (2024). Recent advances in DDAH1 inhibitor design and discovery: insights from structure–activity relationships and X-ray crystal structures. RSC Advances.

[31] Schrodinger L (2015). The PyMOL molecular graphics system. Version.

[32] Takei K, Kanamori H, Nakayama A, Chiba M, Takei Y, Seike I (2023). Screening for metallo-beta-lactamases using non-carbapenem agents: Effective detection of MBL-producing Enterobacterales and differentiation of carbapenem-resistant Enterobacterales. Antibiotics.

[33] Abdel-Halim MS, Askoura M, Mansour B, Yahya G, El-Ganiny AM (2022). In vitro activity of celastrol in combination with thymol against carbapenem-resistant Klebsiella pneumoniae isolates. The Journal of Antibiotics.

[34] Bashir D, Thokar MA, Fomda BA, Bashir G, Zahoor D, Ahmad S (2011). Detection of metallo-beta-lactamase (MBL) producing Pseudomonas aeruginosa at a tertiary care hospital in Kashmir. Afr J Microbiol Res.

[35] Alizadeh H, Khodavandi A, Alizadeh F, Bahador N (2021). Resistance profiling of metallo-betalactamase genes in clinical isolates of Enterobacteriaceae: Emergence of multidrug resistance. Gene Reports.

[36] Tarashi S, Goudarzi H, Erfanimanesh S, Pormohammad A, Hashemi A (2016). Phenotypic and molecular detection of metallo-beta-lactamase genes among imipenem resistant *Pseudomonas aeruginosa* and *Acinetobacter baumannii* strains isolated from patients with burn injuries. Archives of Clinical Infectious Diseases.

[37] Cox S, Harvill L, Bullock S, Smith J, Bergman J (2022). Validation of a method for pantoprazole and its sulfone metabolite in goat plasma using high performance liquid chromatography. Journal of Chromatography Open.

[38] Abdulaal WH, Alhakamy NA, Asseri AH, Radwan MF, Ibrahim TS, Okbazghi SZ (2024). Redirecting pantoprazole as a metallo-beta-lactamase inhibitor in carbapenem-resistant *Klebsiella pneumoniae*. Frontiers in Pharmacology.

[39] Miyajima Y, Hiramatsu K, Mizukami E, Morinaga R, Ishii H, Shirai R (2008). In vitro and in vivo potency of polymyxin B against IMP-type metallo-β-lactamase-producing *Pseudomonas aeruginosa*. International journal of antimicrobial agents.

[40] Smith JR, Rybak JM, Claeys KC (2020). Imipenem-cilastatin-relebactam: A novel β-lactam–β-lactamase inhibitor combination for the treatment of multidrug-resistant gram-negative infections. Pharmacotherapy: The Journal of Human Pharmacology and Drug Therapy.

[41] Castanheira M, Rhomberg PR, Flamm RK, Jones RN (2016). Effect of the β-lactamase inhibitor vaborbactam combined with meropenem against serine carbapenemase-producing Enterobacteriaceae. Antimicrobial Agents and Chemotherapy.

[42] Zhao D, Li H, Yue C, Sun K, Dai Y, Zhang H (2021). Captopril potentiated meropenem activity against MBL-producing carbapenem-resistant *Klebsiella pneumoniae*: in vitro and in vivo study. Journal of Inorganic Biochemistry.

